# Accessing gap-junction channel structure-function relationships through molecular modeling and simulations

**DOI:** 10.1186/s12860-016-0121-9

**Published:** 2017-01-17

**Authors:** F. Villanelo, Y. Escalona, C. Pareja-Barrueto, J. A. Garate, I. M. Skerrett, T. Perez-Acle

**Affiliations:** 1 Computational Biology Lab. Fundación Ciencia & Vida, Santiago, Chile; 2 Centro Interdisciplinario de Neurociencia de Valparaíso, Universidad de Valparaíso, Playa Ancha, Valparaíso, Chile; 3 State University of New York (SUNY) Buffalo State, Buffalo, NY 14222 USA

**Keywords:** Connexins, Hemichannels, Gap-junction channels, Structure and function, Molecular simulation, Homology modeling

## Abstract

**Background:**

Gap junction channels (GJCs) are massive protein channels connecting the cytoplasm of adjacent cells. These channels allow intercellular transfer of molecules up to ~1 kDa, including water, ions and other metabolites. Unveiling structure-function relationships coded into the molecular architecture of these channels is necessary to gain insight on their vast biological function including electrical synapse, inflammation, development and tissular homeostasis. From early works, computational methods have been critical to analyze and interpret experimental observations. Upon the availability of crystallographic structures, molecular modeling and simulations have become a valuable tool to assess structure-function relationships in GJCs. Modeling different connexin isoforms, simulating the transport process, and exploring molecular variants, have provided new hypotheses and out-of-the-box approaches to the study of these important channels.

**Methods:**

Here, we review foundational structural studies and recent developments on GJCs using molecular modeling and simulation techniques, highlighting the methods and the cross-talk with experimental evidence.

**Results and discussion:**

By comparing results obtained by molecular modeling and simulations techniques with structural and functional information obtained from both recent literature and structural databases, we provide a critical assesment of structure-function relationships that can be obtained from the junction between theoretical and experimental evidence.

## Background

Gap junctions (GJs) are regions of cellular membranes in which transmembrane proteins belonging to adjacent cells are in close contact, thereby forming hydrophilic dual-membrane channels. These channels allow the exchange of nutrients, metabolites, ions and small molecules up to ~1 kDa. GJ channels (GJCs) are formed by the end-to-end docking of the extracellular portion of two hemichannels (HCs) or connexons [1] each HC being composed of an hexagonal array of connexins (Cx) protomers [2]. GJCs have crucial roles in many processes including differentiation, neuronal activity, development, immune responses and cell synchronization. Moreover, several human diseases are caused by mutations in connexins, including neurodegenerative diseases, skin diseases, deafness and developmental abnormalities [3].

### From rough to fine: the early ages of GJC structure

**Fig. 1:**
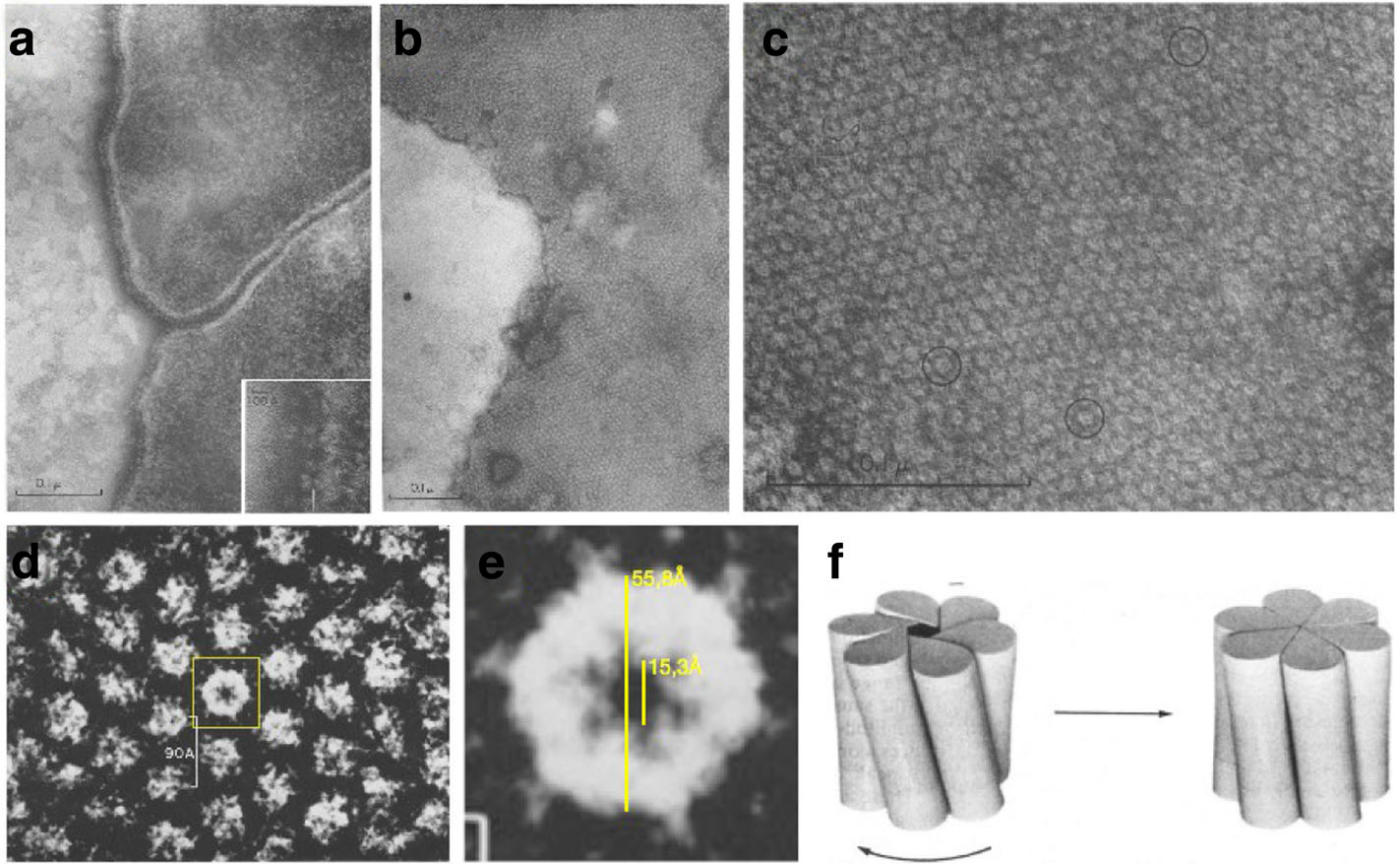
Early electron microscopy images of gap junction channels. **a** Cellular membrane between two rat liver cells. **b** Cellular membrane of rat liver cells immediately after isolation exhibiting the characteristic hexagonal pattern. **c** A highly magnified portion of the cellular membrane shown in Panel **b**. **d** Higher magnification and rotation of a portion of the cellular membrane shown in Panel **c**. **e** A digital zoom-in to the central yellow box denoting the extracellular portion of a hemichannel. Note the clear hexagonal symmetry. **f** The open/close model of a GJC proposed by Unwin and Zampighi [11]. Panels **a** to **e**, adapted from Benedetti and Emmelot [6]. Panel **f**, adapted from Unwin and Zampighi [11]

The discovery of GJs began with the seminal work of Robertson who described them as regular and hexagonal lattices filling the gap between the cellular membranes of adjacent cells [4, 5]. Benedetti and Emmelot [6] described gap junctions as being particularly abundant in regions related to cellular communication and through electron microscopy of rat liver cells, demonstrated their icosahedral structure and hexameric symmetry (Fig. 1, panels A to E). In 1967 Revel and Karnovsky [7] presented findings that led to the term “*Gap Junctions*”. Payton et al., [8] conducted experiments demonstrating that GJ transport molecules. Through the next decade, the Goodenough lab made substantial progress by identifying the chemical components of GJs as common lipids and one specific (yet unidentified) protein [9]. Further studies led to the characterization of the constituent protein of gap junctions, named connexin [10]. It was later proposed that one connexon crossed each junctional membrane forming an interconnecting channel from the cytoplasm of one cell to the cytoplasm of the other, spanning the 2 nm gap between the apposed cells [1]. In this way, the term connexon was used to represent the half-channel (hemichannel) contributed by each cell to create the gap junction channel between cells.

By studying the structure of isolated GJs using high-resolution EM up to 18 Å, Unwin and Zampighi [11] demonstrated that observed hexameric structures formed regular lattices through the cellular membrane. Attention to the electronic density led to their proposal that GJC’s could exist in at least two different states; open and closed. Later, Unwin and Ennis [12], proposed that the GJC state was sensitive to the concentration of Ca^2+^+, and that these divalent cations promoted the switching between the two states of the channel. Therefore, it was established for the first time that GJCs could not only transport solutes but regulate the permeability between cells.

A topological comparison between the HC of α and β connexins emerged from the work of Yeager and Gilula [13] who used anti-peptide antibodies directed to different sites of the protein sequence, together with cleavage by an endogenous protease and 2D-EM imaging. In doing so, these authors proposed a 2D-plot denoting the significant difference between the carboxyl terminal region of α (Cx43) and β (Cx32) connexins. Importantly, despite this sequence divergence, both connexin subfamilies share their quaternary structure, denoting a similar HC architecture. In 1994, Zhang and Nicholson [14], used a similar approach to determine the topological model of Cx26, demonstrating that it is consistent with the previously deduced topology for Cx32 and Cx43. Shortly afterward, in 1997, Unger and colleagues [15] achieved a structural projection of a Cx43 GJC at 7Å resolution revealing that a ring of transmembrane (TM) alpha-helices flanked a central hydrophilic pore and a second ring of alpha-helices was in close contact with the membrane lipids.

**Fig. 2:**
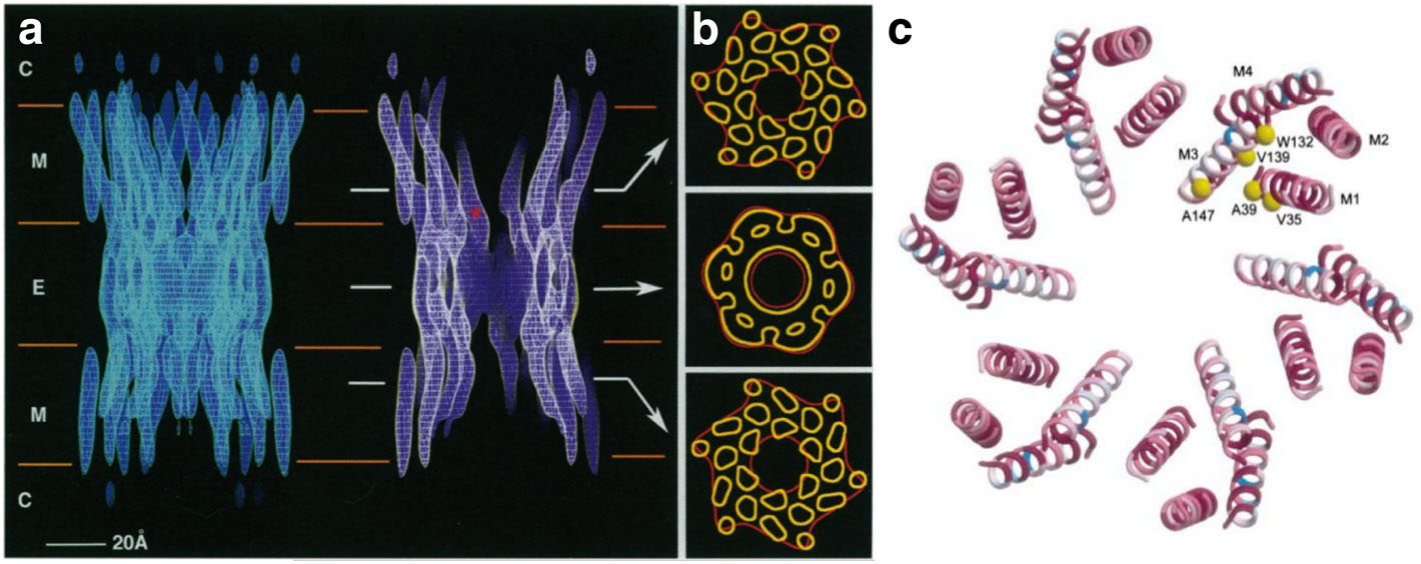
Early gap junction structures determined by electron crystallography and modelling. **a** A 3D EM-derived map of a Cx43 GJC. **b** The densities at different positions show clearly the 24 TMs, four for each monomer. **c** Model of Cα atoms derived by Fleishman et al., [21], showing in yellow the residues identified as pore lining. Panel **a**-**b** adapted from Unger et al., [16]; Panel **c** adapted from Fleishman et al., [21]

The first 3D structure of a GJC was determined by Unger and colleagues in 1999 [16] (Fig. 2a) using Cx43. Using electron crystallography at 7.5 Å resolution, they confirmed that the channel was formed by the end-to-end docking of two HC, exhibiting 24 internal electron densities that were consistent with an alpha-helical conformation of the four TM domains of each connexin (Fig. 2b). However, it was not possible to assign unequivocally the correspondence of each TM in sequence with the observed densities. This fundamental work was in agreement with most of the accumulated structural and functional studies [17] and provided a foundational structural source for gap junction biologists.

In the following years, many studies were designed to obtain more detailed structural information about GJCs and also to relate structural features with transport and gating processes. Methods involving electron cryomicroscopy (cryo-EM) [18], X-ray diffraction [19], atomic force microscopy (AFM) [20], computational methods [21, 22] and nuclear magnetic resonance (NMR) [23] combined with mutational, biochemical, and functional studies (reviewed in [2]) have provided a wealth of information about GJCs.

### The devil is in the details: the race for a high resolution model

The use of computational algorithms in conjunction with the experimental evidence became a promising way of obtaining a more detailed structure of GJC. Fleishman and colleagues [21] provided the first model of the Cα atoms of a GJC based on an improved cryo-EM map and biochemical experiments (Fig. 2c). In this work, the main challenge was the assignment of the TM helices to the corresponding densities in the EM map. Fleishman used a mixed approach of sequence analysis and experimental data. In detail, the sequence analysis was based both on the conservation and side chain polarity of the residues in each TM, with the following constraints: charged and conserved residues at sites of interhelical interaction; less conserved residues facing the lipids or lumen of the pore. They also employed experimental data on accessibility of each region in GJC, revealed using SCAM (substituted-cysteine accessibility method). This technique was carried out by several groups on several GJC isoforms which not necessarily were in agreement, in fact studies on hemichannels identified the pore lining domain as TM1 [24, 25] while studies of GJC’s reported the pore-lining as TM3 [26]. So, in the Fleishman work [21], the pore-lining regions were defined as TM1 and TM3 in a rigid-helix structure with a parallel orientation.

A key aspect of GJC structure is related to the interactions between the two connexons of apposing cells. The EM-maps gave little information about this region and the flexible nature of the extracellular domains. In 2007, Kovacs and colleagues [27] studied the extracellular region of GJC, modeling the interaction as a highly ordered β-barrel-like structure.

Oshima et al. [18] computed 3D maps from EM of a Cx26 M34A mutant. The M34A mutation was employed to create a more stable channel based on information that amino acid substitutions at position 34 result in channels that appear to be stabilized in a partially closed conformation [28]. Oshima et al. [18] observed the general structure of Cx26 channel as similar to the Cx43 channel observed at a resolution of 7.5 Å [16]. In addition, they observed a plug-like density in the pore lumen and proposed that the M34A mutation could generate a conformational change around the N-terminal domain, causing it to block transport, posing as a plausible, explanation for how alterations in function relate to structure. To explain the blocking mechanism, Oshima et al. [18] superimposed the Fleishman et al. [21] Cα model on their EM-map and argued that the flexibility of the N-terminal domain would allow it to enter to the pore acting as a plug only for M34A mutant due to the smaller side chain of alanine in position 34 [18]. This was consistent with biochemical data showing that sidechain length at position 34 is a key determinant of channel function [29].

In 2008, Pantano et al. [30], built an all-atom model of a Cx32 connexin based on TM assignments of Fleishman et al. [21]. In this exhaustive work they rebuilt the side chains using molecular dynamics they fitted the Cα structure obtaining a stable system embedded in a palmitoyl-oleoyl-phosphatidyl-choline (POPC) bilayer. The method introduced in this paper, to reconstruct a protein starting from the protein backbone, was validated on a different channel (the KcsA potassium channel, Pantano et al., [30]). To date, several protein structure have been modeled using this approach [31].

### A light in the shadow: the crystallographic structure of the human Cx26

**Fig. 3:**
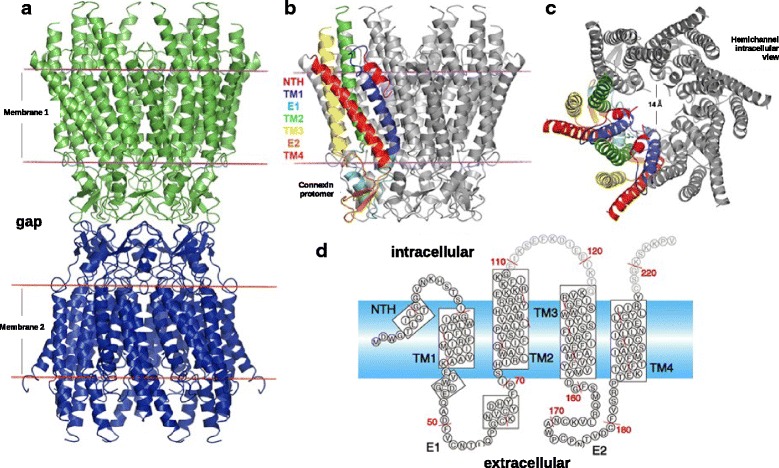
Schematic representation of the basic structural biology of connexins. **a** Secondary structure representation of a gap junction channel formed by end-to-end docking of two hemichannels of Cx26, colored in green and blue. Membrane planes depicted by red solid lines. **b** Representative hemichannel invoving hexameric arrangement of connexin protomers. Protomer domains appear denoted using color-coded names in one protomer as reference. **c** Intracellular view of a human Cx26 hemichannel in its open conformation with inner pore diameter of 14 Å (see text for explanation on channel openness). **d** 2D-plot denoting connexin secondary structure and the approximate position of every residue. The 3D coordinates of the human Cx26 gap junction and membrane planes were retrieved from Orientation of Proteins in Membrane Database [109] (using PDB id: 2ZW3). Panels **a** to **c** were rendered using Pymol. Panel **d** was modified from Nakagawa et al. [32]

Less than a year after the publication of the all-atom model [30], a 3D structure of human connexin 26 (hCx26) was determined at a resolution of 3.5 Å by X-ray crystallography, providing key structural information about connexins GJCs, including identification of the pore lining regions of hCx26 [19, 32] (Fig. 3). The crystal structure demonstrates that each protomer is composed by four transmembrane helices (namely TM1 to TM4), two extracellular loops (E1, E2) and a N-terminal helix (NTH), forming a typical four-helical bundle in which any pair of adjacent helices are antiparallel. The arrangement of the tertiary structure of hCx26 is as follows: NTH, TM1 and TM2 face the pore; TM3 and TM4 are located within the perimeter of the hemichannel facing the membrane lipids, with E1 and E2 are facing the extracellular environment. Thus, this crystal structure confirmed that TM1, NTH, E1 and, to a lesser extent TM2, are the major pore-lining regions of each connexin subunit, discarding any role of TM3 in this function. Regarding inter-protomer interactions, the Cx26 crystal structure shows that they are mostly located in the extracellular half of the transmembrane helices TM2 and TM4, and in the extracellular loops. Residues included in the inter-protomer interactions, as well as those involved in the intra-protomer interactions, are highly conserved among several members of the connexin family, suggesting that there is a conservation of both the protomer folding and the oligomerization process to form the hemichannel, within the connexin family members [33]. Moreover, this structure shed light on an important unresolved issue: the TM helix assignment. This issue arose because the connecting loops between the transmembrane alpha helices were not revealed in the first map published by Unger et al. [16]. In fact, reference to Fig. 4 of Unger et al. [16] noted that with an in-plane resolution of 7.5 Å, there was ambiguity in assigning the molecular boundary of the connexin subunits. The helix assignments in the model of Fleishman et al. [21], which served as a starting point for several other modes [18, 30] are inconsistent with the arrangement of TM helices in the atomic model of Maeda et al. [19].

**Fig. 4:**
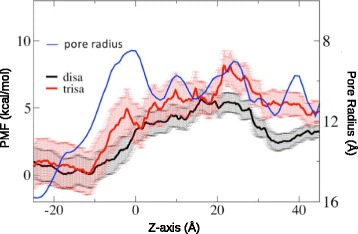
Potential of Mean Force (PMF) as a function of pore length for permeating maltosaccharide solutes compared with pore radius. Taken from Luo et al., [90]

Following this work, a deeper analysis of the crystallographic structure [34] led to heightened focus on the role of the amino terminal helix (NTH) as a key element in channel blockage and gating. New structural information revealed details including a circular hydrogen bond network between the Asp2 side chain and the NTH main chain. Moreover, Trp3 from the NTH and other residues form a hydrophobic patch along the pore lining side of TM1 of the counter-clockwise adjacent subunit. This hydrophobic patch comprises residues M34, V36 and V37. Remarkably, a set of deafness-associated mutations has been mapped to this patch, such as V37I, M34A, M34T and A40V, suggesting that this zone is highly relevant for Cx26 function (reviewed in [35]).

### One template for all: when the models make progress

The crystallographic hCx26 structure not only revealed the detailed structure of a GJC, it opened the door to the development of comparative models using this structure as a template. This is especially important because there are as many as 21 connexin isoforms expressed in different species and different tissues (Harris, [2]). Moreover, each six-protomer connexon can form homo- or heteromeric GJC creating an almost limitless space for structural and functional specializations [36, 37].

Comparative modeling takes advantage of a known 3D structure to build the structure of a different but related (at least in terms of sequence similarity) protein. Early work in the 1980’s [38, 39] and 1990’s [40, 41] demonstrated that protein structure is more likely conserved than sequence, so sequences with at least 40% identity could have very similar structures. Therefore, one sequence can be modeled using the structure of another if they share at least 40% sequence identity. In the case of membrane proteins, this statement is more general, because in transmembrane regions a high structural correlation occurs even when sequence identity is as low as 20% [42].

A key step in the comparative modeling procedure is the sequence alignment between the template structure and the target sequence. This alignment guides the progressive building of the main chain for the target sequence. Each software employs a distinct method (or a combinations of these) such as rigid-body assembly, segment matching or satisfaction of spatial restraints [43]. Modern comparative models are developed using alignments that consider not only protein sequence but also some structural features, for instance the position of the secondary structure elements.

However some groups [32, 44] used a more manual approach to build their models of Cx32 GJC. They started from a multiple sequence alignment without considering structural features and manually corrected the mismatched residues on the Cx26 structure with the corresponding residue on the target Cx32. The side chain structure of these mismatched residues were built with the software COOT [45] and checked for spatial restrictions with the software Procheck [46]. The model of Cx32 was used to generate hetero Cx32-Cx26 GJC and was used to explain their experimental observations related to channel docking. In their work they analyzed inter-connexon interactions in the extracellular space and they identified hydrogen bond networks on the E1-E1 and E2-E2 interfaces, inferring that the latter probably play a pivotal role in docking [44].

The above procedure was also applied to analyze the parahelix of Cx50 [47], a region between TM1 and EC1 that forms an imperfect 3.10 helix in the crystallographic structure [19, 48]. This region seems very stable in several simulations and structure-function studies revealed that it is involved in voltage gating [49, 50]. Tong et al. [47] analyzed the parahelix structure using comparative modelling focusing on charge-changing mutations in this region. They found that local surface electrostatic charges in this region play an important role in the ion permeation.

After main-chain modeling, further refinement can include addition of amino acid side chains. Many programs are capable of using a library of rotamers calculated from experimentally known structures, and selecting according to criteria such as steric clashes, energy considerations, and main chain structure dependency among others [51]. This step is particularly important because the crystallographic structure derived by Maeda et al., [19] lacks the start methionine (M1) and sidechains of several residues. The existence of loops not present in the template structure represents a challenge for most of modeling works as well, and it can be solved via searching for segments that fits on fixed-backbone endpoints, or by a conformational search (ab initio) or by a combination of both [52].

### When the dance begins: the role of simulation in revealing molecular motions

During the last 20 years, computational techniques have become an indispensable tool for understanding of processes at the nanoscale level. The number of articles using comparative models of GJC are increasing in number, with many based on the atomic model of Cx26 [19]. However, the intrinsically dynamic nature of GJC function – to transport molecules from one cell to another – presents a challenge to the analysis based on the static models and the static X-ray structure itself.

Among the methodologies belonging to the field of molecular simulation, molecular dynamics (MD) is widely used for the study and characterization of molecules at an atomic resolution [53]. Starting from an experimentally determined structure, or even from a model [54], MD techniques allow the researcher to “capture” the natural motion of the molecular system and to monitor its dynamic behavior through time. MD deals with the numerical solution of the *N*-body problem raised by Isaac Newton in the 17th century, by elegantly combining statistical mechanics and classical physics, through the time-dependent integration of Newton’s equation of motion. These equations, even in the simplest systems, are of such complexity that the integration must be done with numerical methods over a huge quantity of discrete time steps, instead of being performed in an analytical, continuous fashion. At each time step, the coordinates of the atoms, are used to calculate the potential energy of the system (*V*) and the force. The latter is calculated using a molecular mechanics force field (FF) or potential energy function. A long series of these calculations allows the generation of a trajectory through phase space, defined by the three atomic spatial position and momentum arrays, as representative of a statistical mechanical ensemble of the molecular microstates. In this way, the reliability of MD simulations fully depends on two factors; a) the capacity to explore all regions of phase-space, also known as the sampling problem and b) the ability to faithfully reproduce the potential energy surface of the studied system, the scoring problem [53]. The sampling problem has been partially alleviated by consistent software development, together with advances in specialized computer hardware, as evidenced by recent simulations in the order of hundreds of microseconds [55], moreover special enhanced-sampling techniques can be employed [56]. On the other hand the scoring problem entirely depends on the quality of the FF, which are specifically tailored to a given set of molecular species. Regarding biomolecules, these are commonly termed biomolecular FF, with many “flavors” such as the GROMOS [57], OPLS [58], CHARMM [59], and AMBER [60] force-fields. Even though their parameterization philosophy differs, they are functionally similar. They generally include terms that describe major bonded (bonds, angles, and dihedral angles) and non-bonded (van der Waals and electrostatic) interactions. Parameters used for these energy terms derive from a combination of experimental data and quantum mechanical calculations, tuned to optimally reproduce the structure and vibrational modes of the molecular systems of interest, as well as their thermodynamic properties [61].

Normally the configurational part of a MD is of special interest, because it is possible to analyze atom movements and conformational changes. Thus, the trajectory of a MD i.e., the ensemble of snapshots of atomic coordinates in function of time, is critical for the GJC analysis. The trajectories in MD range from picoseconds to hundreds of nanoseconds, but in the case of GJC they should be long enough to allow such a massive system to adopt the conformational equilibrium required and overcome the sampling problem. The crystallographic structure in Maeda et al. [19] has ~9,800 atoms, the model with completed regions absent in the previous structure and the corresponding hydrogen atoms has nearly 30,000 atoms, moreover the model embedded in bilayer surrounded by solvent (water plus ions) has ~178,000 atoms. So the time needed to get the structure equilibrated from its “frozen” state in the initial structure to the desired temperature structure that mimic the experimental conditions, is near the microseconds time scale [62].

Over the last few decades, MD has been used in the study of a wide range of biological phenomena, such as protein folding, ion conduction, and muscle elasticity [63–66]. The rapid increase of the computational power and recent developments in simulation software have enabled MD studies of significantly larger systems, such as the ribosome in complex with a protein-conducting channel (2.7 million atoms) [67] and of much longer processes (hundreds of microseconds) [68]. In particular, our research group has been using MD simulations since 2003, enhancing understanding of applied and pure research areas such as protein engineering [69], computer based drug design [70, 71] structure and function relationships of GPCRs [72, 73], protein-binding domains in plants [74], viral recognition and hypersensitivity response in plants [75], and water permeability properties of archaeal aquaporins [76]. Despite the enormous success of MD protocols, an important gap remains between the time scales currently accessible by MD simulations and the length of manyl biological processes. As a matter of fact, rotational and translational motions of large-scale domains such as those involved in channel gating (microseconds to milliseconds or even longer), are still far from the reach of traditional MD methods. Furthermore, the intrinsic barriers that exist between important microstates hinders proper configurational sampling, indeed statistical mechanics precludes such barrier crossing when these are high above thermal energy (k_B_T), normally higher than 2.5 KJ/mol (0.6 Kcal/mol) at 300 K. To bridge this gap, special simulation procedures, termed enhanced sampling techniques have been developed to allow the exploration of these slow degrees of freedom [77, 78].

Even though the enhanced sampling techniques have partially alleviated the sampling problem, many biologically relevant processes are above the scope of current atomistic models, consequently simplifications, normally in the form of a drastic reduction of degrees of freedom are employed. In this way, a common approach is to remove unimportant degrees of freedom, normally solvent molecules employing the Brownian Dynamics (BD) formulation [79]. In BD, the dynamics of molecules of interest is controlled by the Potential of Mean Force (PMF), an energy function that includes the averaged effect of the neglected particles, a friction factor and a stochastic term that mimics the effect of random collisions. Proper BD simulations, therefore require the careful parameterization of the PMF term, which can be obtained by rigorous free energy calculations in all-atom MD. BD simulations have been successfully applied in biomolecular simulations of protein crowding and ion-conduction in membrane channels under electro-osmotic gradients, among others [80–82]. In particular for BD simulations of ion-channels, the electrostatic term of potential equation is replaced by a linearized solution of the Poisson-Boltzmann equation. These calculations are numerically costly thus, it is common practice to simulate the channel as a rigid body, impeding the study of gating phenomena due to structural rearrangements. This is the case in the work of Kwon et al. [22] where BD is used to simulate the ion flux considering a fully rigid protein coupled to an ion bath via the Grand-canonical Monte Carlo (GCMC) technique. In GCMC [83], apart from the typical randomized translational, rotational and orientational moves typical of standard Canonical Monte Carlo simulations, random insertions (from a virtual reservoir) or deletions of particles (ions, in this case), via the Metropolis-Hastings algorithm are performed [84, 85]. The probability of insertion/deletion will also depend on the chemical potential of the studied species. Upon usage of 2 baths kept at different chemical potential values, a concentration gradient can be established, rendering an ionic flux, thus current, against the gradient. This mixed approximation allowed calculation of current versus voltage relationships. Otherwise the molecular dynamics imposes a methodological problem in the form of the periodic simulation condition.

The periodicity of a system in molecular simulations assures that any atom reaching one side of the simulation box can appear at the opposite side. This property renders a periodic system that can be considered infinite in any direction of the simulation space. Without periodicity, the atoms would continuously hit the “border” and this would influence some macroscopic properties of the system, like volume and pressure and would render simulations far from reality. The problem with transport-through-channel systems is that the solvent molecules (i.e. ions), prefer to move from one side of the bilayer to the other through the energetically free path the periodic box offers instead of through the channel (i.e. connexin pore).

A simple approximation to study the transport of molecules through channels is the application of an external force to push (or pull) the molecule or molecules to go through a specific path. For ion transport, this force could be applied to the entire system, in the form of a constant electric field parallel to the pore axis, giving rise to a voltage difference along the membrane. This external force can be calibrated to provide the necessary work for the molecules to move from one side to the other according to the direction and magnitude of the applied field. This is the case in the work of Zonta et al., [86] where potentials of −80 mV and 80 mV were applied to study the binding of calcium ions to the extracellular region.

Another possibility of external force usage, is the so-called steered molecular dynamics (SDM) technique. This technique consists of applying an external force to a specific molecule to pull it into a certain direction. The pulling is not exerted directly over the molecule, instead is applied through a virtual spring with a certain stiffness, attached to the atom (or atoms) of interest. The force applied could be constant and generate variable velocity during the movement or be handled by the software to maintain a constant velocity in the selected direction. The latter is commonly used because the force necessary to maintain a constant velocity must counterbalance the forces exerted by the molecular environment (i.e. the pore internal walls) and this could be registered as a function of the distance of movement, in the form of force exerted along the pathway, in other words the work or PMF. With this information the free-energy landscape along a specific reaction coordinate could be analyzed (reviewed in [87]).

For GJC, SMD have been employed to explore the transport of chloride and potassium [86, 88], molecules such as calcein [89] and simple saccharides [90]. In the case of atomic ion transport through gap junctions, a region within the pore opposes their permeation, forming an energetic barrier, the parahelix (PH) region. This region is the second narrowest in the pore, after the NTH region, and is located between TM1 and E1. Interestingly, the NTH region is more flexible and does not provide a high resistance to the passage of solutes, while the PH region is more stable and has a higher number of conserved charged residues, in which induce a very stable electrostatic network that can interact with the studied solutes [48]. In the case of molecules, Luo et al. [90] argue that there is a difference in the energetics of transport for molecular solutes and atomic ions. Molecules have more degrees of freedom, generating substantial entropic barriers as the molecule potentially adopts a specific conformation in order to pass the narrow regions of the pore. On the other hand, for atomic ions, the main contributor to free energy of permeation should be enthalpic due to the lack of internal degrees of freedom of these species and the presence of charged residues within the channel. In this way, Luo et al., [90] analyzed the transport free-energy of two aminopyridyl-labebeld maltosaccharides, one being permeant to GJC (maltose, a disaccharide) the other being impermeant to GJC (maltotriose, a trisaccharide). Briefly, they did not find significant differences in the PMF profiles along the pore pathway (see Fig. 4) and noted configurational entropy as the key to discrimination between solutes. The trisaccharide is bigger, thus more flexible (with more degrees of freedom) and it is therefore less probable that the molecule will adopt the correct conformation to pass through the narrow pore. The disaccharide needs less energy to overcome this conformational barrier and pass through the pore. Nevertheless, proper entropy calculations need to be performed to confirm this claim.

**Fig. 5:**
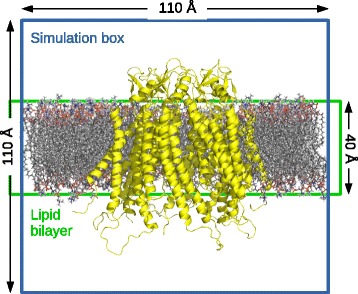
Schematic representation of a typical HC-GJC system simulated in MD. The HC is shown in cartoon representation in yellow. The dimensions are approximated, they could differ in different simulations. Simulation box is typically filled with solvent molecules, i.e. water and ions, not shown for simplicity. Figure prepared with software Pymol

When generating structural models of HCs or GJCs, the explicit representation of lipid bilayers and water molecules provides a more realistic environment for the study of this complex system. Despite the limitations in simulation length due to the number of atoms, MD has been successfully employed to study a wide range of biomolecular systems and phenomena similar in complexity to GJCs [91]. In simulation models employing MD simulations, the system consists of the completed HC embedded in a phospholipid bilayer plus solvent on each side of the bilayer, inside a simulation box of around 1331 nm^3^ [22, 48, 86] (Fig. 5). In all cases, the initial system is energy minimized to avoid steric clashes and after velocities assignment (from Maxwell-Boltzmann’s distributions) an equilibration dynamics and final production dynamics are performed. Due to the presence of a lipid bilayer and to allow the natural fluctuations of the membrane, this simulations must be performed with the NPT ensemble (constant particle number, pressure and temperature) via coupling the system to a virtual thermostat and barostat with anisotropic cell fluctuation, the latter is achieved by scaling velocities and positions, respectively.

### Not all that glitters is gold: revealing the controversies still present after the crystallographic structure

Upon the release of an atomic model of Cx26 using X-ray crystallography [19], some of the controversies surrounding GJC structure seem resolved. However, this structure is a glimpse of a channel composed of one of over 20 different connexin proteins, captured in one state by the crystallographic process at a resolution which is not accurate enough (3.5 Å). In reality there are more unanswered questions than ever. Some of the key questions relate to dynamics properties, in this way, molecular simulation techniques arise as proper tools to generate hypotheses based on structure-dynamics-function relationships encoded in the molecular architecture of the Cx26 GJC.

One of the key questions related to the Cx26 structure [19] involves whether the channel was captured in the open or closed state. When the structure was published, it was speculated to represent an open configuration [19, 34] due to the unobstructed path from one side of the pore to the other. A proposed closed state of the channel was believed to have been presented in the low-resolution EM-map derived in Oshima et al., [18] using the Cx26M34A mutant. This mutant which displayed a distinctive density in the cytoplasmic side of pore lumen, alleged to be the NTH [34] which was not observed in the high resolution atomic model. Therefore the configuration in the crystallographic structure was identified as being in an open state.

**Fig. 6:**
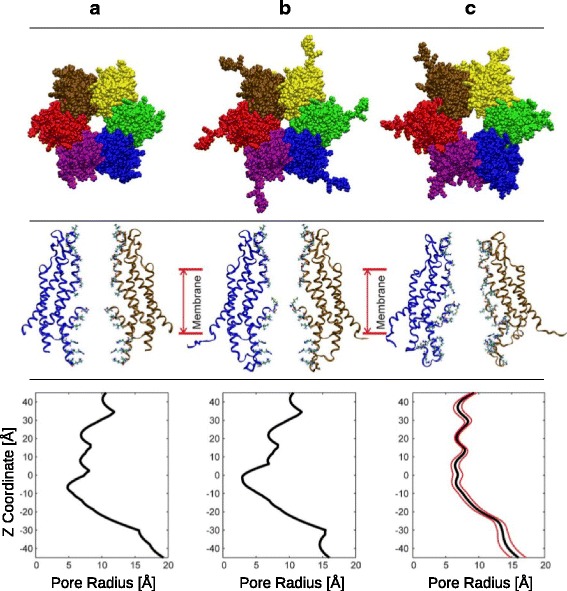
Structure of hCx26. Column (**a**) crystallographic structure [19]; Column (**b**) modeled structure with completed CL and C-terminus plus missing residues and sidechains; Column (**c**), modeled structure after MD simulation. *Upper row*, structure of the channel viewed from extracellular side. Middle row,cartoon representation of two opposing monomers in a side view, with pore-lining residues in ball & stick. Lower row, pore radius as a function of pore length. Taken from Kwon et al., [22]

The above issue was partially addressed by the Bargiello lab using molecular dynamics simulations of the Cx26 hemichannel [22, 48] A goal of the first study involved adding and refining regions not well resolved in the atomic model [19] including the cytoplasmic loop (residues from 100 to 124), the C-terminus (residues from 218 to 226), the initial methionine residue and the side-chain associated with residues K15, S17 and S19. In their first simulation, Kwon et al. [22] used ion-flux calculations via grand-canonical monte-carlo brownian dynamics simulations (GCMC/BD) of this structure and showed that the pore region was too narrow to account for the experimentally observed currents. Under a transmembrane potential, these simulations predicted a marked inward current rectification with an almost full anionic selectivity, in full disagreement with experimental evidence [92]. Maeda et al. [19] reported a minimal pore diameter of 14 Å but Kwon et al. [22] argued atom diameters, missing residues and sidechains such as Met1 were not considered in the calculation (Fig. 6a). The pore diameter after correction is on average, 10 Å [22]. It is important to mention that the simulations employed herein did not account for any structural re-arrangement due to the protein being simulated as a rigid body. So this first simulation served to assess, the “openness” of the atomic model presented by Maeda et al. [19]. A second simulation was performed on the completed structure, constructed by taking the atomic structure [19] revised to include side-chains and loops via comparative modelling. This new structure had an internal pore diameter less than 6 Å (Fig. 6b). It was further refined via long MD simulations leading to a slightly increased pore diameter (Fig. 6c) that matched much more closely the single-channel conductance derived from patch clamp experiments [92].

Another important feature lacking in the x-ray structure is related to co- and post-translational modifications (PTM) identified experimentally that change the net charge of several residues [93]. These are the neutralizing acetylations of the N-terminus and internal lysines K15, K102, K105, K108, K112 and K116. Consequently, Kwon et al., [22] carried out MD simulations including these modifications, which remarkably reproduced the experimentally derived current versus voltage (I/V) relations and cation selectivity. The authors concluded that the MD relaxation of the complete structure plus the charge neutralization derived from co- and post-translational modifications were essential for the development of a model of the open conformation that accounts for the cationic-selective nature of Cx26, and closely resembles the experimental single-channel I/V curves [22].

In a further study, the authors performed extensive MD simulations specifically to explore the structural determinants that stabilize the open conformation [48]. Briefly, they explored the interaction network of the parahelix, which has been suggested as the loop permeability barrier [50]. From energetic analyses of their simulations, it was found that an extensive van der Waals and electrostatic network stabilized the parahelix. Interestingly, this electrostatic network showed a certain level of correlated motion with an adjacent subunit, suggesting a cooperative effect. Consequently, the authors hypothesized that the disruption of this electrostatic network by an external voltage could allow the parahelix to protrude the channel, in a concerted mechanism that would eventually close the pore [48].

Additional disagreement with the atomic structure of Cx26 [19] arose from molecular dynamics simulations of Zonta et al., [86]. The Cx26 GJC structure was simulated using the atomic model of Maeda et al. [19] with completed regions, similar to the aforementioned work [22]. The structure of Cx30 GJC was also modeled and analyzed. The authors confirmed the stability of TM regions, but observed a displacement of NTH towards the TM1, widening the pore lumen. They detected the hydrophobic interactions of Trp3 as described by Maeda [19] but not the hydrogen bonding network around Asp2. The transport process was also analyzed, and the electrostatic interaction networks around the parahelix was described as a potential barrier to ion passage, specifically the residues K41 and E49 in Cx26 and Cx30 respectively [86].

### An old dancer comes to play: the effect of calcium on GJC function

The effect of calcium on GJC function was identified more than 30 years ago [12] but still presents a challenge for those aiming to understand its complex role in GJC function. Extracellular calcium is capable of blocking the voltage-dependent opening of hemichannels, and therefore it is expected to bind to specific amino acid residues facing the extracellular region of connexons [94–96].

A first approximation to model calcium binding came from Zonta et al. [86]. Four Ca^2+^ atoms were introduced into the extracellular space, and different membrane potentials were simulated by applying an external electric field. With an electric field mimicking a membrane potential of −80 mV, these ions interacted with residues E42, D46, E47 and E50 located in the PH-EC1 region. The authors hypothesized that the binding of calcium ions in the PH region could cause the physical occlusion of the pore around this narrow sector. At zero field conditions (no electric field applied) the calcium ions lose the interaction and a field of +80 mV completely abolishes the interaction.

In further studies the nature of the binding was analyzed at a molecular level [97]. Specifically the proposed PTM γ-carboxyl-glutamate on residues E42 and E47 [93] was explored as a candidate for calcium ion binding. This PTM have been previously identified as a possible calcium coordinating moiety. This analysis was followed by quantum chemistry (QM) calculations at the density functional theory level. In QM, not only the atomic cores but the electronic degrees of freedom are simulated, thus rendering these type of techniques quite computationally costly, allowing the simulation of a only a few hundred atoms [98]. QM simulations take a completely different theoretical approximation to simulate atomic motions. This technique takes a quantum mechanics approach, simulating not only the atomic cores but the electronic degrees of freedom. These more realistic considerations are computationally costly because they imply a much higher level of calculation, allowing simulation of small systems, or even sub-systems as in the aforementioned case of γ-carboxyl-glutamate binding of calcium ions [97]. The analysis rendered a plausible mode of Ca^2+^ gating involving structural rearrangements induced via the ion’s interaction with PTM residues, and disruption of a proposed salt bridge network. The analysis rendered a plausible mode of binding of Ca^2+^.

**Fig. 7:**
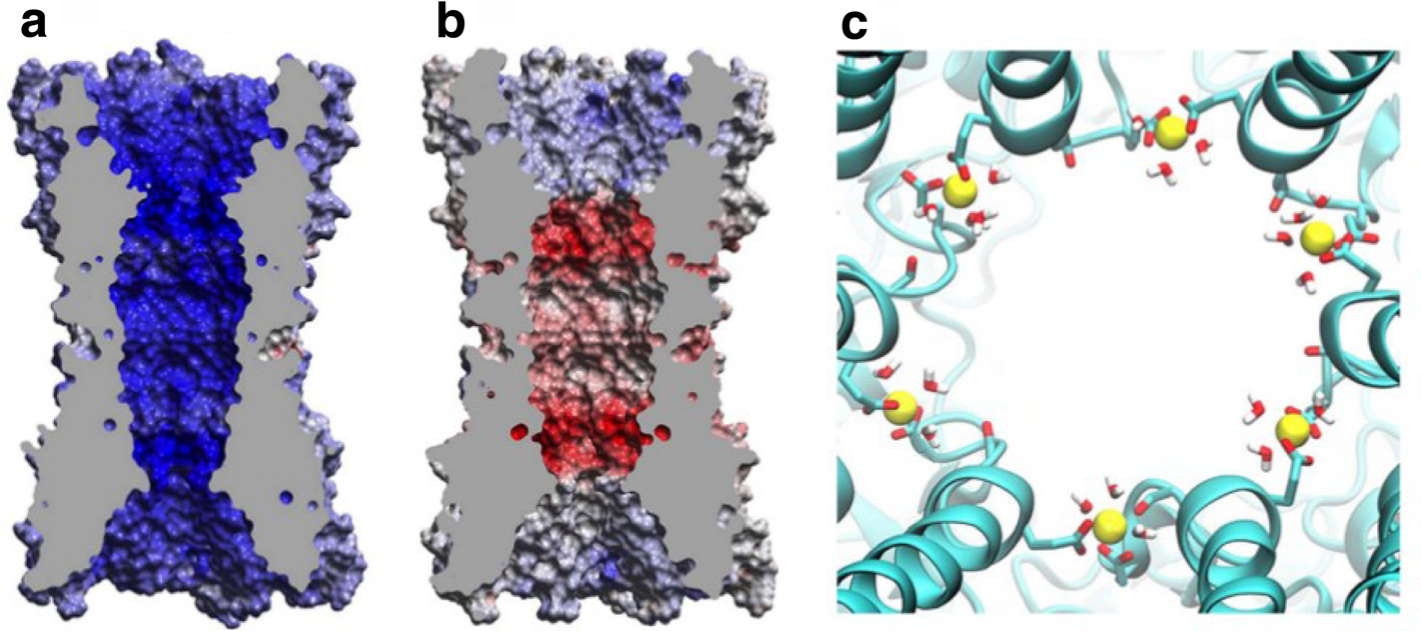
Electrostatic potential surface on crystallographic structures of Cx26 (**a**) Ca^2+^ -bound and (**b**) Ca^2+^ -free. **c** Extracellular view of the interior of the Cx26 pore, highlighting the calcium binding site at the boundaries of the parahelix (PH). Taken from Bennett et al., [99]

In 2016, the structures of both a free and calcium-bound Cx26 GJC were determined at resolutions of 3.8 and 3.3 Å respectively [99]. These structures (Fig. 7) bear strikingly similar to each other and also to the crystallographic structure of a Cx26 GJC [19]. The Ca2+ ions are bound to the PH region, with five coordination atoms forming a square pyramidal geometry (Fig. 7c). These atoms belong to the carboxylate moiety of residues E42 and E47 plus the main-chain oxygen of G45 on the next protomer. This structure – with the exceptions of one salt bridge between E42 and R75 – is nearly identical to the calcium-free structure. Any conformational difference between them is localized to the Ca2+ binding site. The pore lining residues and inner diameter of the pore are not altered by Ca^2+^ binding. This discovery was unexpected because Ca^2+^ blocks the channel and it was presumed that a significant conformational change would be induced by the binding of calcium ions in this very narrow part of the pore. To uncover some dynamic behavior of the structures, MD simulations were performed confirming the stability of calcium binding, with the exception of a G45 main-chain interaction that was potentially unstable. The authors concluded that the mechanism of calcium block (particularly for potassium conduction) is more related to an electrostatic barrier imposed by the positive charge of calcium (Fig. 7) than a structural occlusion due to conformational changes [99]. Recent work in our lab recently revealed that the charge arrangement within a GJC-generic model is crucial for its cationic selectivity [100].

Unfortunately the Cx26 structure doesn't resolve the position of all the residues, and the authors didn't discuss the proposed PTM in the same E42 and E47 residues identified as calcium binding sites [93, 97]. However this work is an interesting effort to unveil the structural role of calcium and the mechanism of conductance blockade, and more work in the field of molecular simulation will likely provide more progress in the area.

### Finding the lost water: the discovery of the hCx26 IC pocket

**Fig. 8:**
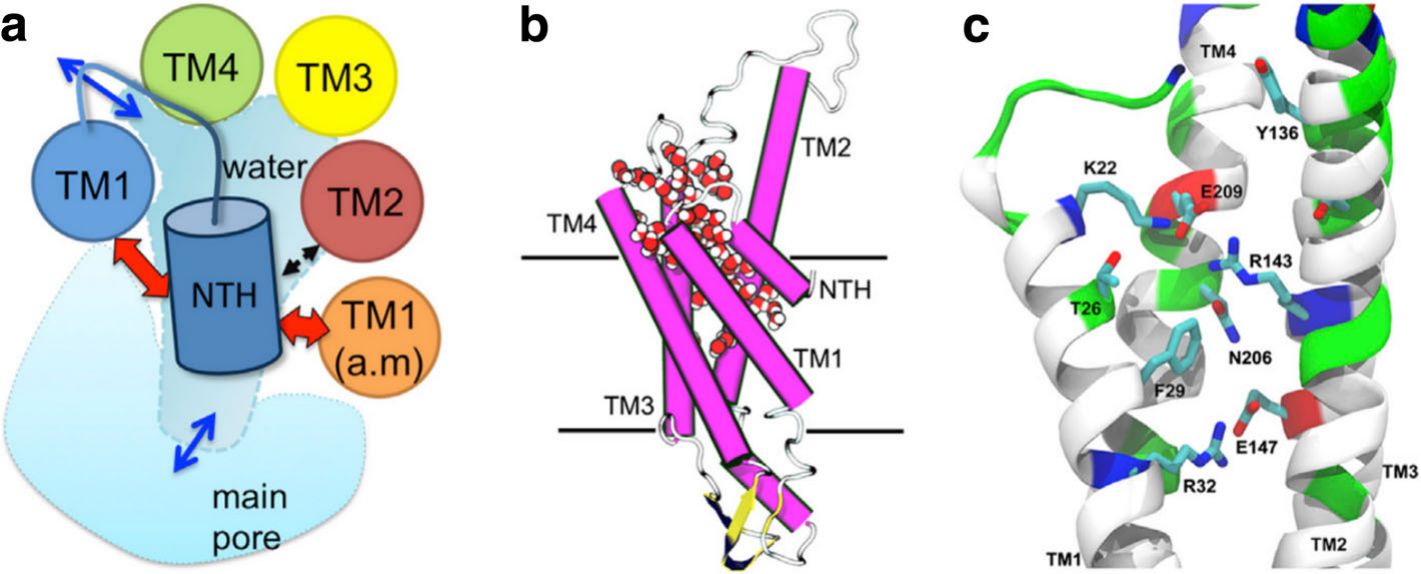
The IC pocket of hCx26. **a** Schematics of IC pocket localization in one monomer, viewed from *top*. **b** Localization of the IC pocket between NTH, TM2, TM3, TM4 and TM1, denoting water inside the pocket using van der Waals representation. **c** Amino acid residues composing the IC pocket represented using sticks and colored by atom type. For clarity, the channel has been rotated 180° on the vertical axis with respect to (**b**) and the NTH removed. Taken from Araya-Secchi et al. [101]. Following the convention of Maeda et al. [19], the intracellular membrane face is located at *top*

About 3 years ago our group initiated molecular modelling studies of GJCs beginning with a study of Cx26. Molecular dynamics simulations of the completed structure of Cx26 HC (with PTMs) offered an excellent source of information about connexons and their transport processes. While studying the dynamic behavior of the NTH and its possible role in the slow gating process, a cavity was found between NTH, TM2 and TM3 in each of the six protomers (Fig. 8) [101]. This cavity was filled with water molecules from the solvent (Fig. 8a), and phylogenetic analysis demonstrated that amino acid residues facing this cavity are highly conserved. Moreover, by conducting comparative molecular modelling the cavity - termed the IC pocket - was detected in all selected representatives of GJC (Pareja et al., unpublished work). The IC pocket is consistent with accessibility of side-chains revealed by substituted cysteine accessibility method (SCAM) that led to the identification of TM3 as the GJC pore lining [26]. In SCAM analysis of Cx32 GJCs, accessible residues were identified in all TM domains including five residues in TM1 and six residues in TM3 (Fig. 8b). Of the five residues in TM1, only one (133C) faces the proposed water pocket. The others lie along the pore-facing region of TM1 (I30, F31, M34 and V35) facing the NT in the crystal structure of Cx26. Interestingly, these were accessible only in a proposed closed state of the channel. Of the six residues in TM3 that were accessible to sulfhydryl reagents, three correspond to residues facing the IC pocket (Y135, S138 and V139). Other accessible residues were located toward the extracellular end of TM3 (F141, L144 and F149) and a proposed mechanism of access to these residues has not been formally proposed.

Protein cavities, in particular those filled by water, play significant biological roles in transmembrane proteins. They are known to influence the activation/deactivation events of GPCRs [102], the ligand-binding processes of cannabinoid and beta-adrenergic receptors [73, 103, 104], intermolecular recognition events [105–107] and protein folding [107, 108]. By analyzing water dynamics it was determined that the water molecules within the IC pocket are different from bulk water, having a slower dynamic behavior and a longer residence time. Moreover, the number of water molecules in the IC pocket is correlated with the orientation of residues R143 and F29 (Fig. 8c). The former forms an electrostatic network that changes depending on the IC pocket state and could play an important role in channel activity. Interestingly, NTH orientation was related to changes in the electrostatic network involving R143 which influences IC water occupancy. The NTH orientation was measured as distance from protein center of mass, or as angle with pore axis [101]. In vitro functional assessments of hCx26 and charge-altering mutants at position 143 were conducted by evaluating the uptake of molecular tracers through HCs. The results demonstrated that R143 is a key residue regulating conductance of hCx26 HCs to tracer molecules [101].

## Conclusions

Many challenges to better understanding gap junction structure and regulation are related to the complex nature of these large intercellular channels. The complexity of a full GJC embedded in a two membrane system makes these channels difficult to purify and even more difficult to crystallize for x-ray diffraction experiments. This explains the scarcity of crystallographic structures - only three in 50 years. It also explains regions of poor resolution in the existing structural models [19, 99].

In functional analyses, the challenge of working with intercellular channels has been partially circumvented by analysis of HC. These channels are amenable to electrophysiology and reconstitution and have provided a wealth of information about channel gating, regulation and permeability [2]. However, more work is needed to understand gating and regulation of GJC.

Other key questions are related to the mechanism by which disease-related mutations affect the channel, and this can be answered by comparing mutants with the Cx26 structures [19, 99]. Other questions include; what are the mechanisms of GJC gating, is there a structural correlation between cytoplasmic and extracellular portions of the pore and, what are the determinants of transport and permeability. Other important questions are related to the changes in sequence and structure that result in a diverse array of GJCs.

Although the current crystallographic structures are rigid, frozen snapshots of the channel, they have provided an astonishing amount of information and an irreplaceable starting point for simulation studies. These simulations unveil the dynamic behavior of GJC channels, further refine key regions in the structure, and model conformational changes that may occur in response to stimuli such as solute passage or applied voltage. Their use will undoubtedly lead to a better understanding of GJ channel structure and function, particularly when performed in conjunction with experimental techniques.

Molecular simulation, e.g. MD, can be considered as the *in silico* version of a conventional microscope – *the (numerical) molecular microscope* – with the advantage of providing a live view of the biomolecule at an atomic resolution. Time-averaged properties that are computed from an MD trajectory can be compared with macroscopic quantities, which are averages over time and multiple copies, that are measured experimentally. Furthermore, molecular simulation techniques offer the opportunity to explore systems under an unlimited number of artificial conditions that are often inaccessible experimentally. For instance, a residue can be changed to mimic the effect of an experimentally studied point mutation, or even neutralize the electrostatic forces generated by any given group of atoms.

The advance in processing power and availability of computational hardware offer more capacity for simulations and extend simulation times offering the ability to explore explicitly the conformational space. The use of advanced sampling techniques, for instance replica exchange with swarms of trajectories, may be the next step in obtaining more detailed models of GJC function.
